# A Hybrid Model Based on a Dual-Attention Mechanism for the Prediction of Remaining Useful Life of Aircraft Engines

**DOI:** 10.3390/s25185682

**Published:** 2025-09-11

**Authors:** Chenwen He, Zixiang Li, Chenyu Zheng, Zikai Zhang, Liping Zhang

**Affiliations:** 1 Key Laboratory of Metallurgical Equipment and Control Technology of Ministry of Education, Wuhan University of Science and Technology, Wuhan 430081, China; hcw18372724254@163.com (C.H.); z173570209@163.com (C.Z.); zhangzikai@wust.edu.en (Z.Z.); zhangliping@wust.edu.cn (L.Z.); 2 Hubei Key Laboratory of Mechanical Transmission and Manufacturing Engineering, Wuhan University of Science and Technology, Wuhan 430081, China; 3 Precision Manufacturing Institute, Wuhan University of Science and Technology, Wuhan 430081, China

**Keywords:** remaining useful life prediction, transformer, attention mechanism, temporal feature extraction block, sensor feature extraction block

## Abstract

Estimating the Remaining Useful Life (RUL) of aircraft engines plays a vital role in the field of prognostics and health management. In multi-dimensional time series regression tasks, accurately capturing both time series features and sensor features, as well as integrating these two types of features, poses a significant challenge for RUL prediction. The sensor features represent the weights of each sensor on the RUL prediction results. To overcome this challenge, we introduce a hybrid model based on a dual-attention mechanism. Initially, a temporal feature extraction block is applied to map the time-step dimension into a hidden representation space, facilitating the capture of complex temporal dynamics. These patterns are then refined using a multi-head self-attention mechanism. Subsequently, a sensor feature extraction block is applied to capture sensor-specific characteristics. Each sensor sequence is treated as a separate channel, compressed to derive sensor weights, and integrated to form global features that fuse temporal and sensor-level representations. Finally, RUL is estimated via a regression layer. The proposed method is demonstrated to be effective on the Commercial Modular Aero-Propulsion System Simulation (C-MAPSS) dataset. Compared with the state-of-the-art CTNet model, the proposed method achieves 7% and 9% gains in RMSE and Score, respectively, on the FD001 dataset.

## 1. Introduction

Prognostics and health management (PHM) is an advanced, integrated framework that encompasses fault diagnosis, RUL prediction, predictive maintenance, and related functionalities [1,2]. Among these, RUL prediction is a critical component, serving as a key indicator of the real-time condition of mechanical equipment [3]. It entails predicting how much longer machinery will function effectively by analyzing data collected from sensors. By analyzing this data, the current degradation state of the equipment can be inferred, allowing for reliable forecasts of its future operational capacity. Such predictions enable the implementation of predictive maintenance strategies, which not only substantially reduce production costs but also mitigate the risk of equipment failures and associated safety risks [4,5].

At present, methods for predicting the remaining useful life (RUL) of mechanical equipment are generally divided into three primary categories: model-based methods, data-driven methods, and hybrid approaches that integrate both paradigms [6,7]. The model-based approach describes the degradation process of a device by constructing physical models or mathematical equations and combining them with relevant empirical knowledge for RUL prediction. These methods are highly interpretable due to their transparency and clear physical underpinnings. However, their applicability to complex systems is often limited by the difficulty of accurately modeling such systems [8]. Data-driven approaches utilize historical operational data, such as sensor data, fault data, etc., to train models and learn device degradation patterns [9]. These approaches eliminate the need for intricate physical modeling and exhibit strong adaptability, but they typically need a large amount of high-quality data and considerable computational resources. Hybrid approaches aim to integrate the transparency of model-based techniques with the adaptability and learning strength of data-driven models. While they offer a promising balance, they are often more complex to design and require careful parameter tuning.

Driven by ongoing advancements in industrial big data and technological innovation, the volume of historical data generated by machinery and equipment has grown substantially. This progress has significantly accelerated the development of data-driven approaches and has attracted growing interest from researchers [10]. A variety of deep learning architectures—including convolutional neural networks (CNNs) [11,12], recurrent neural networks (RNNs) [13,14,15], graph neural networks (GNNs) [16,17], and Transformers [18,19] have been extensively utilized in the field of RUL prediction. These models offer powerful nonlinear mapping capabilities and strong representational learning capacity, providing distinct advantages over traditional model-based approaches.

However, individual models, such as CNNs, RNNs, and GNNs, typically focus on extracting a single type of feature and often fail to simultaneously model both temporal dependencies and inter-sensor correlations [13,20,21]. To overcome this limitation, researchers have designed various model variants that are capable of extracting features across both temporal and spatial dimensions. Nevertheless, despite these advancements, the effectiveness of jointly extracting features from both domains remains constrained [22,23].

The introduction of attention mechanisms (AMs) led researchers to integrate them into CNN and RNN architectures, resulting in moderate improvements in model performance [24,25]. However, these enhancements did not fully address the inherent limitations of CNNs and RNNs. Transformer models have brought notable advancements and have shown strong performance in predicting RUL [26,27]. Unlike CNNs, Transformer architectures substantially enhance the utility of attention mechanisms by enabling more effective global feature extraction [28]. The ability of Transformers to capture extended temporal dependencies makes them well-suited for time-series-based RUL estimation.

Despite their advantages, Transformer models also have limitations. Although they are effective at extracting global features, they often struggle to capture local patterns [29]. This poses a challenge in RUL prediction, where equipment degradation frequently manifests as subtle, short-term changes between adjacent time steps—patterns that Transformers may overlook [29,30]. Integrating customized attention mechanisms allows the model to prioritize adjacent time steps and lessen the impact of more distant ones, enhancing its sensitivity to localized degradation patterns.

AMs have been widely adopted across various domains, including machine translation, time series forecasting, and image recognition [31]. As a typical multidimensional time series regression task, RUL prediction has also benefited from the integration of AMs. Studies utilizing attention for RUL estimation from temporal and feature viewpoints typically fall into two major categories.

The first method focuses on extracting global features. In this strategy, CNNs are employed to capture global representations, followed by attention mechanisms that highlight important features while suppressing less relevant ones [32]. However, this method inherits the limitations of CNNs: although local features are well captured, the method does not sufficiently represent the device’s overall degradation characteristics.

The second approach emphasizes temporal dynamics. Models such as Transformers [33] utilize attention mechanisms to identify subtle variations between time points and learn degradation patterns over time. While effective in modeling temporal dependencies, the standard Transformer architecture does not inherently capture inter-sensor relationships. It tends to focus on the temporal behavior of individual sensors, whereas in RUL prediction, capturing the interactions and correlations among multiple sensors is equally crucial.

Additionally, hybrid models have been widely explored in recent studies. For example, Xiang et al. [34] proposed a single-gated RNN with a differential weighted information storage mechanism, Xiang et al. [35] developed a dynamic self-learning neural network, and Li et al. [36] introduced a channel-independent bidirectionally gated Mamba with an interactive recurrent mechanism. All of these approaches achieved outstanding results in their respective domains. Such models preserve the strengths of their baseline architectures while providing additional advantages. In contrast, the model proposed in this paper integrates two baseline models in series rather than in a hierarchical configuration.

Based on earlier findings, this work presents a dual-attention-based hybrid framework for RUL prediction. The model employs a multi-head full attention mechanism to learn temporal dependencies from sensor data over time, along with an improved channel attention module to assign importance to individual sensors. By combining both attention mechanisms, the model effectively learns temporal patterns as well as inter-sensor relationships. Despite the model’s simple structural design, it achieves high prediction accuracy when evaluated using NASA’s C-MAPSS dataset.

This study offers the following key contributions:

(1) To overcome the limitations of traditional models in feature extraction, the proposed approach introduces distinct attention mechanisms to separately capture temporal and sensor-specific features, thereby enhancing the richness of the learned representations.
(2) For temporal feature extraction, a multi-head full attention mechanism is employed. Specifically, an inverted module from the iTransformer architecture is adopted to allow the model to focus on the temporal behavior of individual sensor sequences while disregarding inter-sensor interference.
(3) For sensor feature extraction, a channel attention mechanism is utilized to learn sensor-specific weights. This study is, to our knowledge, the earliest to implement a channel attention strategy tailored for sensor-wise feature learning in the context of RUL estimation.

The paper will proceed as follows: Section 2 elaborates on the hybrid model framework; Section 3 presents the experiments carried out on the C-MAPSS dataset; and Section 4 concludes the paper with key observations.

## 2. Proposed Methodology

**Figure 1:**
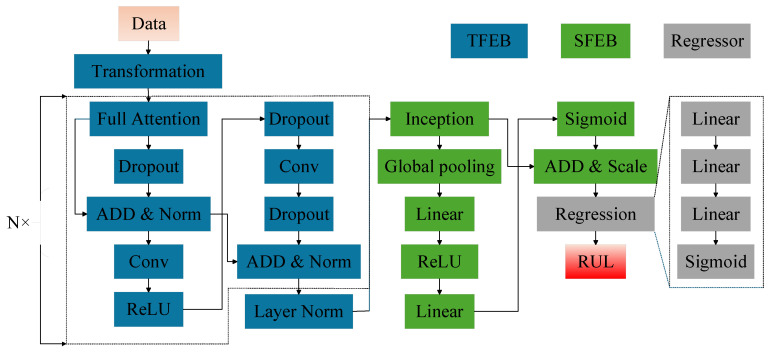
Overall architecture of the HMDAM model.

This section outlines the architecture and design of the proposed Hybrid Model with Dual-Attention Mechanism (HMDAM), developed for predicting RUL from multi-sensor time-series data. As shown in Figure 1, the architecture includes two core components: temporal attention and sensor-specific feature attention mechanisms.

The Temporal Attention Mechanism (TAM) is constructed to uncover hidden temporal relationships within the sensor data. It operates independently on each sensor’s time series, focusing exclusively on intra-sensor temporal patterns without incorporating inter-sensor interactions. This module allows the model to track and learn degradation patterns as they develop over time.

To enhance feature relevance, the feature attention mechanism is used to process the temporal attention block’s output. It captures distinct characteristics from each sensor, allowing the model to assess their individual contributions to system degradation. With a lightweight and efficient structure, the feature attention module integrates sensor-level information while preserving the temporal context learned in the preceding stage.

**Figure 2:**
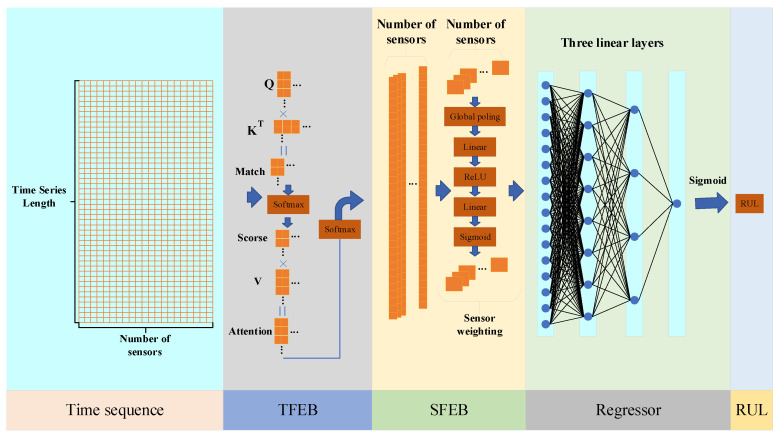
Data processing procedure in the HMDAM framework.

Overall, the HMDAM consists of three integral parts: a temporal feature extraction block (TFEB), a sensor feature extraction block (SFEB), and a regression component for RUL prediction. The TFEB is designed to capture sequential degradation patterns over time, while the SFEB focuses on identifying sensor-specific contributions. The combined operation of these two modules results in a robust and well-rounded feature representation. The regression module then predicts RUL based on the integrated features. The full structural design of the model is depicted in Figure 2. A comprehensive overview of the model’s architecture and its constituent modules is presented in the remaining part of this section.

### 2.1. Time Feature Extraction Block

The TFEB consists of three main components: an embedding layer, the TAM, and a projection layer. The primary objective is to transform raw multi-sensor time-series data into meaningful temporal representations that capture the underlying degradation behavior of mechanical systems.

This module is tailored to detect nuanced variations over time, allowing the model to better grasp patterns associated with system degradation. The process begins with the embedding layer, which performs a dimensional transformation by expanding the time dimension of the input data. This transformation facilitates the extraction of fine-grained temporal features in subsequent stages, while deliberately ignoring inter-sensor correlations at this point to ensure a focus on individual temporal dynamics.

Subsequently, a TAM is employed on the expanded time sequences, allowing the model to focus on the most relevant temporal segments for degradation assessment. This enhances its responsiveness to temporal fluctuations and strengthens its capacity to identify early degradation indicators.

Finally, a projection layer is applied to return the extracted temporal features to their initial dimensional space. This operation maintains consistency in feature representation prior to forwarding the output to the subsequent module in the model.

The architecture and functionality of each individual subcomponent are elaborated in the subsections that follow.

#### 2.1.1. Embedding Layer

The embedding layer performs two essential functions. First, it transposes the input sensor data to facilitate independent time-series feature extraction for each sensor. Second, it increases the dimensionality of the time series, projecting the original temporal input into a higher-dimensional space to enable the extraction of more nuanced temporal patterns. This transposition operation, originally introduced in the iTransformer model, has been shown to significantly enhance the effectiveness of temporal feature extraction [37].

Given the original sensor input data $X=\{x_{1},,,,,x_{t}\}\in R^{T\times N}$, where  T is the number of time steps and N is the number of sensor channels, the embedding layer first transposes the input to obtain $X^{T}\in R^{N\times T}$. A linear transformation is then applied to project the temporal dimension T into a higher-dimensional hidden space of size $d_{model}$. This process is defined as

(1) $$H=Linear(X^{T})\in R^{N\times d_{model}}$$

In Equation (1), $H=\{h_{1},,,,,h_{N}\}\in R^{N\times d_{model}}$, where N is the number of sensors and $d_{model}$ is the dimensionality of the hidden space. Linear denotes a fully connected layer that performs a linear mapping of the transposed time sequences into the hidden representation space.

#### 2.1.2. Temporal Attention Mechanism

The TAM captures time-dependent patterns in the sensor data and converts them into rich, continuous feature representations. TAM consists of a multi-head full attention layer, two-layer normalization modules, and a feed-forward neural network (FFN). It processes the output H from the embedding layer and enhances it by learning temporal correlations within each individual sensor’s time series.

TAM takes the input matrix $H\in R^{N\times d_{model}}$ and passes it through a multi-head full attention layer to capture temporal relationships. Specifically, H is linearly projected into query (Q), key (K), and value (V) matrices as follows:

(2) $$Q=H\cdot W_{Q}$$

(3) $$K=H\cdot W_{K}$$

(4) $$V=H\cdot W_{V}$$

In these equations, $Q,\;K\in R^{N\times d_{k}}$ and $V\in R^{N\times d_{v}}$, where $d_{k}$ and $d_{v}$ represent the dimensions of the projected key/query and value vectors, respectively. Typically, $d_{k}$ = $d_{v}$. The matrices $W_{Q},\;W_{K}\in R^{d_{model}\times d_{k}}$, and $W_{V}\in R^{d_{model}\times d_{v}}$ are learnable parameters.

To improve training stability and speed up convergence, layer normalization is used after the attention step. The FFN is then used to further refine the extracted temporal features, followed by an additional normalization step to maintain consistency in the feature distribution.

Next, the query, key and value matrices are used to compute the scaled dot-product attention. This operation captures time dependency by calculating the attention weights of each sensor at different times. The attention weights are calculated using the following formula:

(5) $$Attention\left(Q,K,V\right)=softmax(\frac{QK^{T}}{\sqrt{d_{k}}})V$$

In Equation (5), the resulting matrix $Attention\in R^{N\times d_{v}}$ represents the learned temporal attention features. The scaling factor $\sqrt{d_{k}}$ is introduced to keep the dot product values within a manageable range, helping to maintain gradient stability during training.

Since TAM employs a multi-head full attention mechanism, it can capture multiple types of temporal dependencies within the time series. Every attention head applies distinct trainable weight matrices to transform the query, key, and value inputs individually. As a result, each head computes a distinct representation of temporal relationships, Allowing the model to capture different facets of the degradation behavior. Each sub-attention module produces an output ${Head}_{n}\in R^{N\times d_{v}}$, where n denotes the index of the attention head.

The combined outputs from all attention heads are transformed via a linear network layer to generate the unified representation of the multi-head attention mechanism. The computations are defined as follows:

(6) $${Head}_{n}=Attention(Q\cdot W_{Q}^{n},\;K{\cdot W}_{K}^{n},V\cdot W_{V}^{n})$$

(7) $$MultiHead\left(Q,K,V\right)=Concat({Head}_{1},\ldots {Head}_{n})W_{O}$$

In these expressions, $W_{Q}^{n},\;W_{K}^{n}{\in R}^{d_{model}\times d_{k}},$ and $W_{V}^{n}\in R^{d_{model}\times d_{v}}$ represent the trainable projection matrices associated with the n-th attention head. The output projection matrix $W_{O}\in R^{(n\cdot d_{v})\times d_{model}}$ is the output projection matrix used to map the concatenated multi-head outputs back into the original feature space.

By attending to multiple representational subspaces across time, the model gains improved awareness of temporal dynamics and generates more expressive feature representations.

After the multi-head attention step, the resulting output undergoes layer normalization to promote training stability and accelerate model convergence. This process is represented as

(8) Y=LayerNorm(MultiHead)

In Equation (8), $Y\in R^{N\times d_{model}}$ r denotes the normalized output and $MultiHead\in R^{N\times d_{model}}$ denotes the result produced by the multi-head attention module.

Next, the normalized output Y is then input into a position-wise FFN, enabling nonlinear mapping to strengthen the model’s feature extraction capabilities. This component enhances the model’s capacity to identify intricate degradation behaviors. The FFN comprises two fully connected layers and is defined as

(9) $$Y_{1}=Linear(Y)\in R^{N\times d_{ff}}$$

(10) $$Y_{2}=Linear(Y_{1})\in R^{N\times d_{model}}$$

In these expressions, $Y_{1}\in R^{N\times d_{ff}}$ is the intermediate representation and $Y_{2}\in R^{N\times d_{model}}$ represents the ultimate result produced by the feed-forward network. The parameter $d_{ff}$ is the hidden dimensionality of the FFN, typically set larger than $d_{model}$ to allow for richer representations.

Finally, the output $Y_{2}$ is combined with the original input Y via a residual pathway, and the result is subsequently normalized through an additional layer normalization process. This operation preserves the original features while enhancing training stability:

(11) $$Y_{3}=LayerNorm(Y_{2}+Y)$$

In Equation (11), $Y_{3}\in R^{N\times d_{model}}$ represents the output produced by the TAM module, which represents the extracted temporal features of the sensor data.

#### 2.1.3. Projection Layer

The projection layer serves to transform the temporal features obtained from the TAM into the same dimensional space as the original input. This is achieved through a linear transformation followed by a transposition operation. The transformation ensures that the learned temporal representations can be seamlessly integrated with subsequent components of the model. The projection operation can be expressed as

(12) $${Y_{T}}^{T}=Linear(Y_{3})\in R^{N\times T}$$

(13) $$Y_{T}={\left({Y_{T}}^{T}\right)}^{T}\in R^{T\times N}$$

In these expressions, $Y_{T}\in R^{T\times N}$ denotes the concluding output of the TFEB. It retains the same shape as the original input sensor data while embedding rich temporal information extracted through the dual-attention processing. This output serves as the input to the subsequent SFEB.

### 2.2. Sensor Feature Extraction Block

The SFEB is designed to enhance the representation of sensor-specific information by integrating it with the temporal features obtained from the TFEB. This module is derived from the SENet model proposed by Hu et al. [38] in 2018. SENet assigns weight coefficients to each channel through lightweight feature extraction. In this work, the SENet-based module is employed to compute weight coefficients for individual sensors. The SFEB consists of multiple layers, incorporating pooling layers, dense (fully connected) layers, and nonlinear activation functions. Its primary objective is to ensure that the final feature representation retains both temporal dynamics and sensor-specific characteristics, which significantly contribute to the reliability of RUL forecasting.

The process begins with a permutation operation applied to the output of the TFEB, denoted as $Y_{T}$, in order to reorganize the data dimensions. Specifically, the single-channel temporal output is reshaped into N distinct channels—each corresponding to a different sensor. This transformation prepares the data for channel-wise processing in the subsequent layers, enabling the model to learn sensor-dependent degradation features more effectively.

Next, the squeeze-and-excitation (SE) operations are applied to each sensor channel to capture sensor-specific importance. This mechanism consists of two main steps.

(1) Squeeze: The temporal data in each sensor channel is compressed into a global feature vector, summarizing the overall temporal behavior of each sensor.
(2) Excitation: A nonlinear transformation is applied to the global feature vector, and attention weights are generated using a sigmoid activation function. These weights are then applied to adjust the original input, thereby enhancing the contribution of important sensor features and suppressing less informative ones.

The process begins with a permutation operation on the output of the TFEB, which rearranges the data for channel-wise processing:

(14) $$Y_{4}=Permute(Y_{T})\in R^{N\times T\times 1}$$

In this expression, $Y_{4}\in R^{N\times T\times 1}$ represents the permuted data, where each sensor is now treated as an independent channel, with its temporal data aligned along the second dimension.

Next, a squeeze operation is applied to each channel to obtain a compact global representation. This is accomplished using global average pooling, which computes the mean value of each sensor’s time series, effectively summarizing its overall contribution:

(15) $$Y_{5}=F_{sq}(Y_{4})\in R^{N\times 1\times 1}$$

In Equation (15), $Y_{5}\in R^{N\times 1\times 1}$ is the output of the squeeze operation. Each element in $Y_{5}$ represents the average temporal value for one sensor, forming a compact representation of that sensor’s overall behavior across time.

Following the squeeze operation, an excitation process is applied to $Y_{5}$ to model the interdependencies among different sensor channels. This step allows the network to adaptively highlight informative sensor features, enhancing the expressiveness of the extracted features. The excitation process consists of three main components: a full connection layer, a ReLU activation function, and another fully connected layer. The computations are as follows:

(16) $$Y_{6}=Linear(Y_{5})\in R^{\frac{N}{r}\times 1\times 1}$$

(17) $$Y_{7}=ReLU(Y_{6})\in R^{\frac{N}{r}\times 1\times 1}$$

(18) $$Y_{8}=Linear(Y_{7})\in R^{N\times 1\times 1}$$

In these expressions, r denotes a reduction ratio that controls the dimensionality of the intermediate layer, effectively reducing computational complexity while retaining representational capacity. The resulting excitation vector $Y_{8}\in R^{N\times 1\times 1}$ contains learned weights that indicate the weight or priority given to different sensor channels.

To obtain the final sensor-wise attention weights, a sigmoid activation function is used to the excitation output $Y_{8}$, producing normalized weight representations for each sensor channel. These weights are then used to rescale the input temporal features $Y_{4}$ through element-wise multiplication, effectively integrating both temporal and sensor-specific characteristics. A final permutation operation restores the data to its original dimensional format. The process is defined by the following equations:

(19) $$Y_{9}=Sigmoid(Y_{8})\in R^{N\times 1\times 1}$$

(20) $$Y_{10}=Scale(Y_{9}⨀Y_{4})\in R^{N\times T\times 1}$$

(21) $$Y_{O}=Permute(Y_{10})\in R^{1\times T\times N}$$

In these expressions, ⨀ denotes element-wise multiplication; $Y_{9}$ contains the learned sensor attention weights; $Y_{10}$ represents the weighted combination of temporal and sensor features; and $Y_{O}\in R^{1\times T\times N}$ is the final output of the SFEB, encoding a fused representation of temporal and sensor-specific characteristics in a format consistent with the original input dimensions.

### 2.3. Regressor

The regressor is responsible for mapping the global features extracted by the TFEB and the SFEB to the RUL prediction. The projection process within the regressor consists of two main steps.

First, the two-dimensional output vector $Y_{O}\in R^{1\times T\times N}$ is reshaped into a one-dimensional vector ${Y_{O}}^{1}\in R^{(T\cdot N)\times 1}$. This reshaping operation allows the subsequent layers to process the global features as a flattened vector.

Next, ${Y_{O}}^{1}$ is subsequently processed through three fully connected layers that progressively reduce dimensionality while improving the model’s capacity to learn robust, generalized feature representations. These layers help capture complex nonlinear relationships in the fused feature space, ultimately enhancing both the precision and reliability of the RUL estimation.

The corresponding mathematical operations are defined as follows:

(22) $${Y_{O}}^{1}=Reshape(Y_{O})\in R^{(T\cdot N)\times 1}$$

(23) $${Y_{O}}^{2}=Linear({Y_{O}}^{1})\in R^{n_{1}\times 1}$$

(24) $${Y_{O}}^{3}=Linear({Y_{O}}^{2})\in R^{n_{2}\times 1}$$

(25) $$RUL=Linear({Y_{O}}^{3})\in R^{1\times 1}$$

In these expressions, ${Y_{O}}^{1}$, ${Y_{O}}^{2}$, and ${Y_{O}}^{3}$ are intermediate representations; {$n_{1}$, $n_{2}$} are the hidden dimensions used during the dimensionality reduction process; and $RUL\in R^{1\times 1}$ represents the final scalar prediction of the RUL.

This composition enables the model to capture a rich mapping from the fused temporal–sensor feature space to the target output, enhancing prediction reliability and the model’s robustness to varying operational patterns.

## 3. Experimental Study

This section presents a detailed evaluation of the developed hybrid model. First, the dataset used in the experiments is introduced, highlighting its characteristics and relevance to the RUL prediction task. Next, the experimental setup, including data preprocessing procedures, model configuration, and evaluation metrics, is described in detail. Finally, we discuss the ablation analysis and comparison of the model against leading methods to confirm its strength in terms of effectiveness, resilience, and adaptability.

### 3.1. Dataset

This study utilized the C-MAPSS (Commercial Modular Aero-Propulsion System Simulation) dataset, a benchmark dataset released by NASA [39]. As shown in Table 1, C-MAPSS is segmented into four sub-datasets—FD001 to FD004—distinguished by varying operational contexts and failure modes. Specifically, FD001 and FD003 each operate under a single condition, with FD001 containing one fault mode and FD003 involving multiple fault types. In contrast, FD002 and FD004 are more complex, featuring several operating conditions and up to six distinct fault modes, which increases the difficulty of accurate RUL prediction.

Each dataset subset includes both a training and testing partitions. The training portion consists of full engine life-cycle data, capturing the complete progression from normal functioning to eventual failure. All engines are equipped with 21 sensors, which continuously monitor various operational parameters such as temperature, pressure, and fan speed.

In this study, the test set was constructed by randomly selecting partial life-cycle sequences from each engine in the training data. These truncated sequences simulate real-world scenarios where the engine has not yet failed, thereby allowing the model to predict the RUL. This configuration allows for a practical assessment of the model’s prediction capabilities across different operating scenarios and patterns of degradation.

### 3.2. Experimental Setting

#### 3.2.1. Data Preprocessing

**Figure 3:**
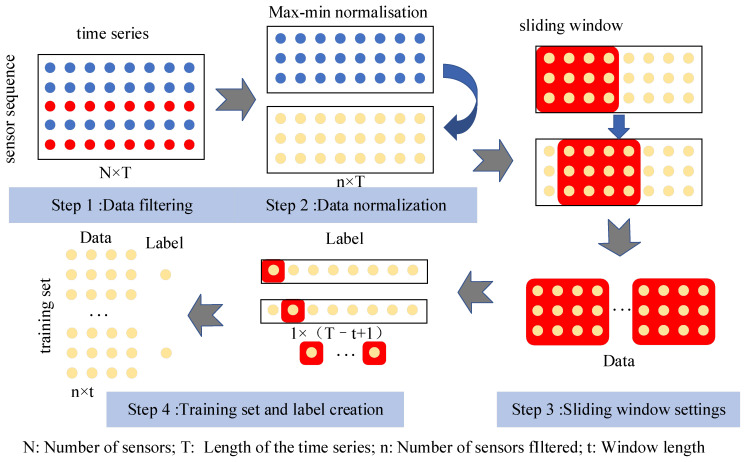
Overview of the data preprocessing procedure.

Prior to experimentation, the raw C-MAPSS dataset requires preprocessing and conversion into a structured format that is appropriate for model training and assessment. The preprocessing pipeline consists of four primary steps: data filtering, data normalization, sliding window configuration, and sample-label construction. The overall preprocessing workflow is illustrated in Figure 3.

(1) Data filtering

The C-MAPSS dataset includes sensor readings from 21 different sensors installed on each engine. These sensors continuously monitor a range of operational and environmental conditions throughout the engine’s lifecycle. However, further analysis indicated that certain sensor readings showed minimal change or remained nearly constant throughout an engine’s operational lifespan. These constant or near-constant signals typically do not contribute meaningful information about the degradation process and may introduce redundancy or noise into the model.

**Figure 4:**
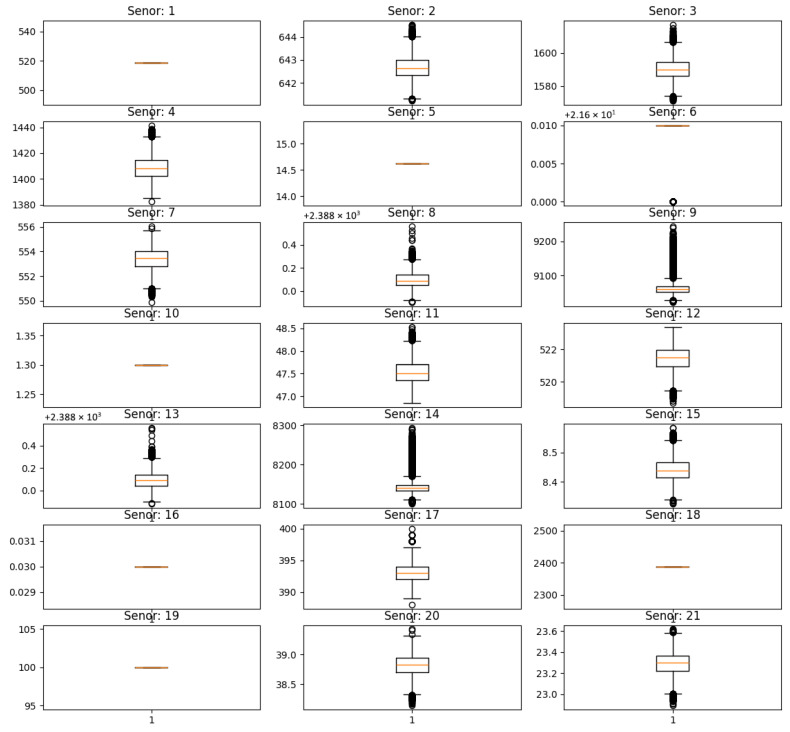
Sensor data distribution in the FD001 dataset.

To ensure more informative and high-quality input data, these uninformative sensors were removed. Using the FD001 subset as a representative case, Figure 4 shows the time-series trends of all 21 sensors. It is evident that sensors 1, 5, 6, 10, 16, 18, and 19 displayed constant or near-constant behavior across all operational cycles. Consequently, these sensors were removed from the feature set utilized in later stages of model development. These seven sensors measured the following physical quantities: total inlet temperature, inlet pressure, total duct pressure, engine pressure ratio, burner air-fuel ratio, set fan speed, and set core engine equivalent speed.

This filtering step ensures that the model focuses only on sensors that carry diagnostic information relevant to the RUL prediction, thereby reducing input dimensionality and potential overfitting.

(2) Data normalization

The sensor readings in the C-MAPSS dataset exhibit considerable variation in both scale and fluctuation range across different sensors. Directly using these raw, unnormalized values can obscure subtle but important patterns—especially for sensors whose numerical values fall within narrow ranges. Such disparities can bias the model toward features with larger magnitudes and reduce its ability to learn meaningful degradation patterns from less prominent signals.

To ensure consistency and eliminate scale-related bias, each sensor’s data was scaled using Min–Max normalization. This approach transforms all sensor readings into a common scale within the range [0, 1], thereby preserving the relative variation patterns while standardizing feature magnitudes.

The normalization method was mathematically formulated and is presented in Equation (26):

(26) $$x_{norm}^{i,j}=\frac{x^{i,l}-x_{min}^{j}}{x_{max}^{j}-x_{min}^{j}}\in [0,\;1]$$

In this expression, $x^{i,l}$ represents the data value recorded by the j-th sensor at the i-th time step; $x_{norm}^{i,j}$ indicates the normalized counterpart of the original value; and $x_{max}^{j}$ and $x_{min}^{j}$ correspond to the highest and lowest recorded values of the j-th sensor, respectively.

Aligning feature scales through this normalization enhances both the stability and efficiency of the training process.

(3) Sliding window settings

To boost the training sample size and better learn short-term temporal patterns, the original time series is divided into overlapping fixed-length segments using a sliding window method. This method is particularly effective in modeling the gradual evolution of system degradation over time.

In this method, a fixed window size is defined, and the window slides across each time series with a stride of 1. At each step, a window of sensor readings is extracted, resulting in a large set of overlapping samples. This dense sampling strategy ensures that subtle degradation patterns are captured from various temporal contexts, improving the model’s ability to learn robust short-term and transitional features.

The benefits of this approach are twofold: it augments the training set by generating multiple samples from each time series, thus mitigating the issue of data scarcity; in addition, this approach maintains the sequential order of data within each segment, enabling the model to capture short-term degradation patterns effectively.

Once the segmentation is complete, all windowed sequences are aggregated to construct the final training dataset.

(4) Training set and label creation

**Figure 5:**
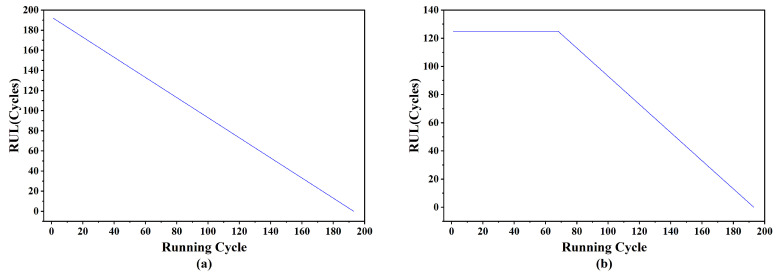
Two degradation patterns of Engine 1 in FD001: (**a**) linear degradation model; (**b**) piecewise linear degradation model.

To generate the RUL labels, the original values are replaced using a piecewise linear strategy that better reflects real-world degradation patterns. As illustrated in Figure 5a, the RUL of an engine naturally declines over time throughout its operational life. However, instead of modeling this decline as a strictly linear decay from the initial time step, a segmented degradation rule is applied.

In particular, the RUL is assumed to remain constant during the early stage of engine operation and begins to decrease linearly only after reaching a predefined time threshold, as depicted in Figure 5b. This approach reflects the practical observation that engines typically operate without significant degradation during the initial phase of their life cycle. In this study, the threshold was set to 125 cycles, consistent with prior work [40].

After preprocessing, the FD001 dataset is transformed into multiple sets of n×t matrices paired with 1×1 labels, where n denotes the window length and t represents the number of selected sensors. These sensors primarily monitor physical quantities such as temperature, pressure, rotational speed, and airflow rate. Finally, the training set is further split for validation purposes. The training data is split by randomly allocating 80% for model training and reserving the remaining 20% for validation. The test set stays fixed to allow an unbiased comparison of model results.

#### 3.2.2. Evaluation Metrics

To evaluate the performance of the proposed approach, three widely used metrics were employed: Root Mean Square Error (RMSE), Mean Absolute Error (MAE), and the Score function. RMSE is a standard metric in regression analysis that quantifies the average magnitude of the prediction errors. MAE is a statistical measure of the average difference between predicted and actual values. They are defined as

(27) $$RMSE=\sqrt{\frac{1}{N}\sum_{i=1}^{N}{\left(\hat{Y_{i}}-Y_{i}\right)}^{2}}$$

(28) $$MAE=\frac{1}{N}\sum_{i=1}^{N}\left|\hat{Y_{i}}-Y_{i}\right|$$

where $Y_{i}$ is the true RUL of the i-th test sample, $\hat{Y_{i}}$ is the corresponding predicted RUL value, and N represents the total number of samples in the test set.

Accurate and timely RUL predictions are crucial in industrial contexts, as delays can compromise safety and disrupt processes. Therefore, the Score function is designed to be more sensitive to late predictions, making it more aligned with the needs of industrial production environments [41]. The Score function is computed using the following formula:

(29) $$Score=\left\{\begin{array}{c} \sum_{i=1}^{n}\left[e^{-\left(\frac{\hat{Y_{i}}-Y_{i}}{13}\right)}-1\right],\;for\;\hat{Y_{i}}<Y_{i}\\ \sum_{i=1}^{n}\left[e^{\left(\frac{\hat{Y_{i}}-Y_{i}}{10}\right)}-1\right],\;for\;\hat{Y_{i}}>Y_{i} \end{array}\right.$$

In Equation (29), $\hat{Y_{i}}$ and $Y_{i}$ denote the estimated and actual RULs, respectively, and n indicates the total number of samples used for testing. In engine degradation scenarios, early predictions are generally more valuable than late ones. Accordingly, the asymmetric structure defined by parameter settings “10” and “13” assigns a higher penalty to late predictions [39].

#### 3.2.3. Training Parameter Settings

To maximize model effectiveness, a comprehensive grid search is conducted to refine the training and structural parameters. This exhaustive search helps identify the best configuration for maximizing prediction accuracy and model stability. The final training hyperparameter configurations are listed in Table 2 with the corresponding structural parameters provided in Table 3.

### 3.3. Analysis of HMDAM

#### 3.3.1. Setting of Sliding Window Size

The window length controls the extent of historical information available to the model along the temporal dimension. If the window is too short, the model cannot capture long-term degradation patterns, resulting in performance loss. Conversely, an overly long window may introduce early-stage noise unrelated to degradation and increase information complexity, which also degrades performance. Therefore, selecting an appropriate window length is critical for achieving reliable predictions. Given the heterogeneity in the C-MAPSS sub-datasets, which was characterized by distinct fault patterns and operating environments, a tailored analysis of sliding window lengths was needed to optimize the HMDAM performance for each dataset.

This study investigated the impact of sliding window sizes ranging from 20 to 50, in increments of 2, with all other parameters held constant during the experiments. Model performance was evaluated using two metrics: RMSE and the S-score. To mitigate the influence of randomness during training, each configuration was run five times for 100 epochs, and the best performance among the five runs was reported as the final result.

**Figure 6:**
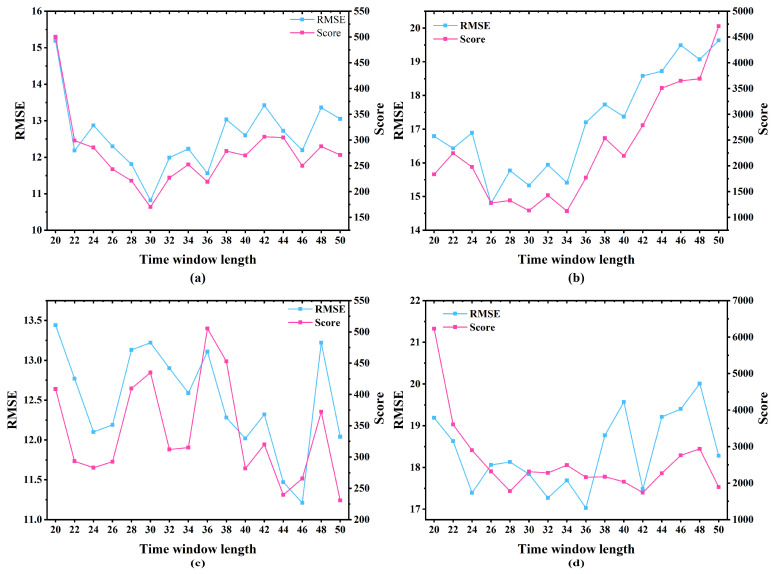
Performance using different time window lengths: (**a**) FD001; (**b**) FD002; (**c**) FD003; (**d**) FD004.

As shown in Figure 6, subfigures (a), (b), and (d) follow the regression line, whereas subfigure (c) exhibits pronounced fluctuations. This behavior may stem from the fact that FD003 contains multiple failure modes, leading to more complex degradation dynamics. In addition, FD003 has fewer samples than FD002 and FD004, which may increase the statistical variance during hyperparameter tuning and, in turn, cause the fitted curve to fluctuate more strongly. As depicted in Figure 6, for the FD001 dataset, both the RMSE and Score reached their lowest values when the sliding window length was 30, making it the optimal choice. In contrast, for FD002, FD003, and FD004, the points with the minimum RMSE and Score values did not coincide. In such cases, the window size associated with the lowest RMSE was selected to ensure better predictive accuracy. Accordingly, the optimal window lengths determined for FD002, FD003, and FD004 were 26, 46, and 36, respectively. To summarize, the best-performing sliding window lengths for FD001 through FD004 were 30, 26, 46, and 36.

#### 3.3.2. Model Parameter Settings

To identify the optimal hyperparameters for the proposed model, a combination of control variable methodology and grid search was employed. Specifically, four key hyperparameters were selected, and four candidate values were assigned to each hyperparameter for the experimental evaluation. The range of values considered for each hyperparameter is presented in Table 4. Model performance was evaluated using two metrics: RMSE and S-score.

**Figure 7:**
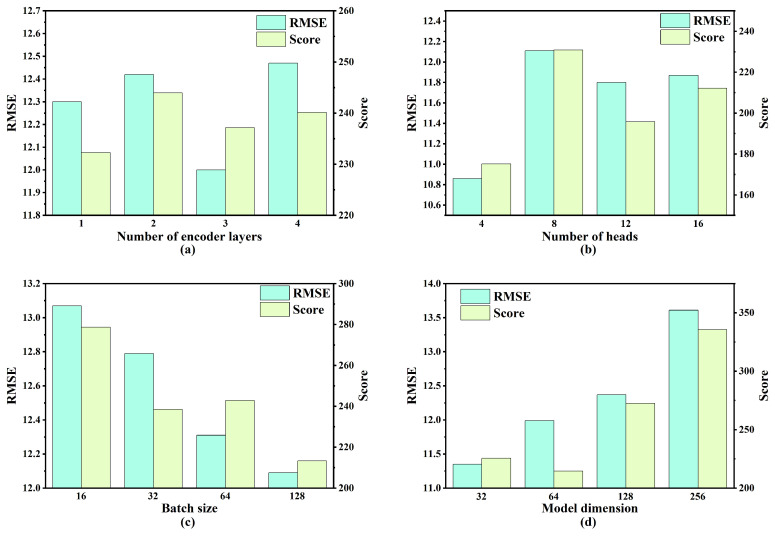
Performance comparison under varying hyperparameter settings for FD001: (**a**) number of encoder layers; (**b**) number of heads; (**c**) batch size; (**d**) model dimension.

**Figure 8:**
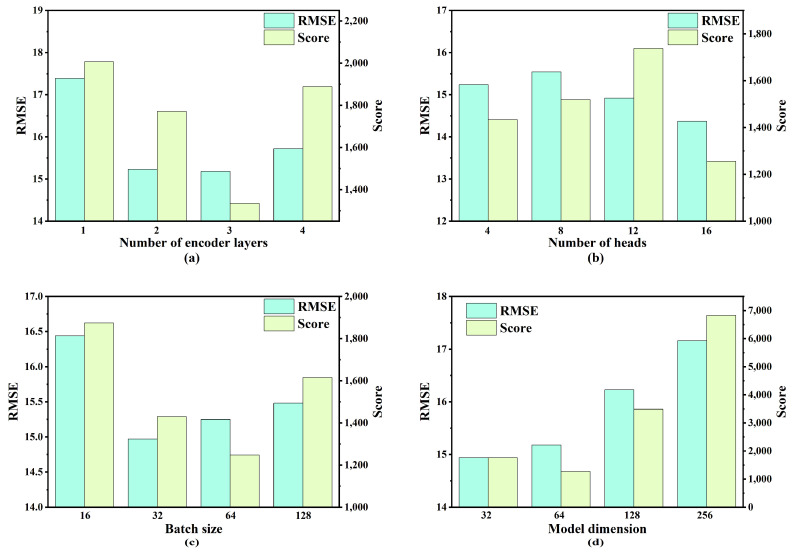
Performance comparison under varying hyperparameter settings for FD002: (**a**) number of encoder layers; (**b**) number of heads; (**c**) batch size; (**d**) model dimension.

**Figure 9:**
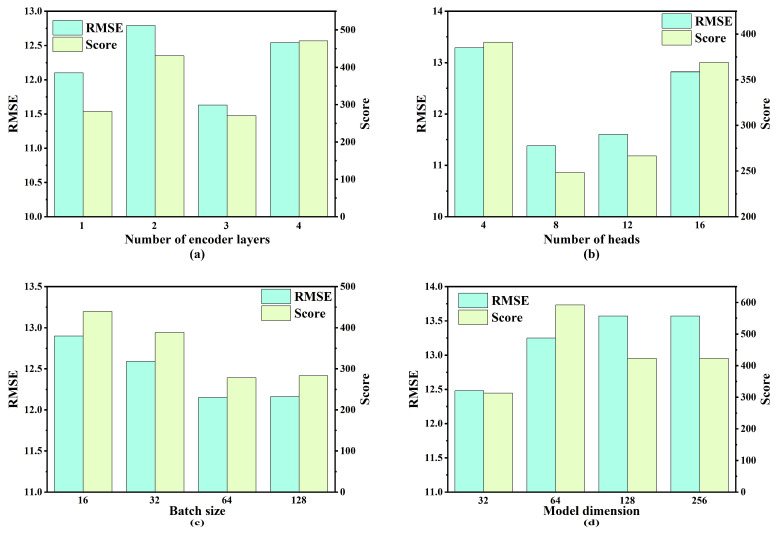
Performance comparison under varying hyperparameter settings for FD003: (**a**) number of encoder layers; (**b**) number of heads; (**c**) batch size; (**d**) model dimension.

**Figure 10:**
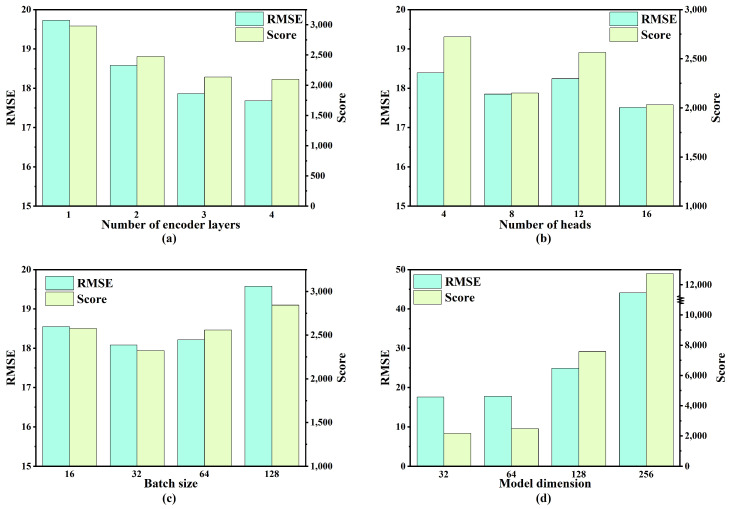
Performance comparison under varying hyperparameter settings for FD004: (**a**) number of encoder layers; (**b**) number of heads; (**c**) batch size; (**d**) model dimension.

As illustrated in Figure 7, Figure 8, Figure 9 and Figure 10, the optimal hyperparameters for the model varied between the different sub-datasets, reflecting the differences in operational conditions and fault modes. The RMSE and Score continued to serve as the primary evaluation metrics. In cases where the RMSE and Score metrics yielded conflicting results, the configuration with the best RMSE value was selected as the optimal setting given its stronger correlation with predictive accuracy.

For the FD001 sub-dataset, the optimal hyperparameters were as follows: three encoding layers, four attention heads, a batch size of 128, and a model dimension of 32. The optimal hyperparameter configurations for the remaining sub-datasets (FD002, FD003, and FD004) are summarized in Table 5.

#### 3.3.3. HMDAM Ablation Experiment

An ablation analysis was conducted to assess the contribution of each individual component in the HMDAM architecture. Three model variants were developed for comparison:

(1) The baseline Transformer model without any enhancements;
(2) The Transformer model equipped with only the TFEB, referred to as iTransformer;
(3) The Transformer model integrated solely with the SFEB, referred to as ST.

Each model was tested under the same experimental setup, with their performance measured using RMSE and the Score function. Following model training, the results—summarized in Table 6—demonstrate the effectiveness of the TFEB and SFEB modules and the overall improvement provided by their integration into the HMDAM.

As shown in Table 6, the Transformer model integrated with the TFEB module (iTransformer) consistently outperformed the baseline Transformer across all four sub-datasets. Similarly, the Transformer model enhanced with the SFEB module also demonstrated improved performance compared to the original Transformer model. Notably, the proposed HMDAM model, which combines both the TFEB and SFEB modules, achieved the best overall performance. The findings demonstrate that both the TFEB and SFEB modules play a vital role in improving RUL prediction accuracy, and their integration leads to complementary enhancements in model performance.

#### 3.3.4. Analysis of Model Results

**Figure 11:**
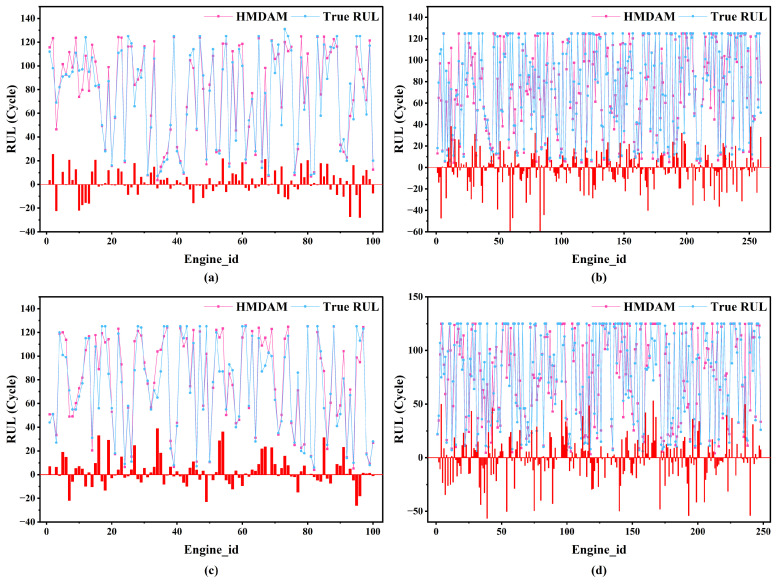
RUL prediction results using the HMDAM model for (**a**) FD001; (**b**) FD002; (**c**) FD003; (**d**) FD004.

After determining the optimal hyperparameters, as outlined in the previous section, the best-performing model configuration was applied to each sub-dataset to predict the RUL using the respective test sets. Figure 11 presents the predicted results, demonstrating a close match between the estimated and actual RUL values for all four sub-datasets. The strong alignment between the predicted and actual values suggests that the HMDAM model delivers high predictive accuracy and demonstrates strong generalization capabilities in estimating the RUL of aero-engine systems.

**Figure 12:**
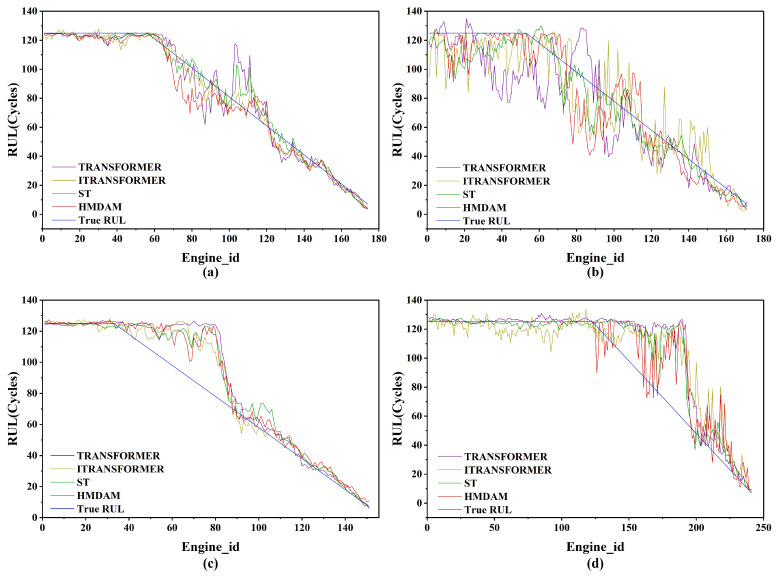
RUL prediction performance for selected engine units across the four sub-dataset: (**a**) the 34th engine in test set FD001; (**b**) the 138th engine in test set FD002; (**c**) the 46th engine in test set FD003; (**d**) the 12th engine in test set FD004.

To further verify the efficacy of the model, several engine units were randomly sampled from each of the four sub-datasets for visual analysis. As shown in Figure 12, the RUL estimates generated by the HMDAM model align more closely with the actual RUL curves than those from the other three baseline models, underscoring its enhanced predictive performance.

Notably, the model maintained strong predictive accuracy during both the initial and final phases of engine degradation. However, some limitations were observed during the mid-stage of degradation, particularly in the FD003 dataset. As shown in Figure 12c, the actual RUL began to decline during this transitional phase, while the model’s predictions lagged behind. This discrepancy is attributed to the complex nature of the mid-stage, which contains overlapping signals from both healthy and deteriorating conditions. The subtle degradation indicators during this period pose a greater challenge for accurate modeling, leading to a temporary reduction in predictive performance.

#### 3.3.5. Model Efficiency Analysis

Using the FD001 dataset as an example, this study compared the four models in terms of FLOPs and parameter counts, with the results summarized in Table 7. the number of FLOPs of the proposed model was 30% lower than that of the ST model and only 1.36% higher than that of the Transformer model. By contrast, the iTransformer achieved markedly lower number of FLOPs, representing just 53% of that of the HMDAM. In terms of parameter counts, the difference between the Transformer and HMDAM was only 2%, while iTransformer’s parameter count was less than 1% higher. Conversely, the ST model required 12% more parameters. These results indicate that the proposed model improves prediction accuracy without substantially increasing the computational overhead, thereby offering a practical solution for real-world RUL prediction tasks under cost constraints.

#### 3.3.6. Comparison with Other Methods

This section presents a performance comparison between the HMDAM model and several state-of-the-art approaches in this domain. The performance outcomes are detailed in Table 8 and Table 9, where the highest scores for each metric are emphasized in bold. These comparisons are intended to highlight the HMDAM model’s effectiveness and competitive edge in predicting RUL under varying operational scenarios.

As shown in Table 7 and Table 8, the Transformer model outperformed the CNN model, while the BGT model surpassed the DCNN model in all metrics. This can be attributed to the fact that CNNs are well suited for extracting local features but have limited ability to capture global dependencies. In contrast, Transformers can effectively model both local and global features when sufficient data and network depths are available [42].

On the FD001 sub-dataset, the HMDAM delivered superior performance, surpassing all the baseline models in terms of both RMSE and Score. Specifically, it surpassed the CTNet model [43], with improvements of approximately 7% in RMSE and 9% in Score, demonstrating its effectiveness in scenarios with simpler operating conditions and failure modes.

Although the HMDAM did not achieve the top rank for all four sub-datasets, it consistently performed at a high level. For the more complex FD002, FD003, and FD004 sub-datasets, the HMDAM model consistently secured second place in the performance rankings, closely following the BGT model [44] and CTNet. These results affirm that the HMDAM maintains strong predictive performance across varying levels of dataset complexity, confirming its robustness and generalizability.

Regarding the Score metric, although the HMDAM model performed well on the FD001 dataset, it lagged behind the BGT and CTNet models on the other datasets. This suggests that the HMDAM has limitations in mitigating RUL overestimation. The CTNet model combines the multi-head self-attention mechanism of Transformers with the cross-channel information propagation of graph neural networks, which increases its complexity [43]. Such complexity enhances its ability to fit complex data, leading to superior performance. By contrast, the HMDAM does not markedly increase FLOPs or the parameter count relative to Transformer models, reflecting its relatively low complexity. This may account for its weaker performance in the Score metric compared with CTNet.

**Table 8:** RMSE comparison between HMDAM and other advanced methods.

Method	FD001	FD002	FD003	FD004	Average
BiLSTM ([45])	13.65	23.18	13.74	24.86	18.86
DCNN ([46])	12.61	22.36	12.64	23.31	17.73
CatBoos ([47])	15.8	21.4	16.0	22.4	18.90
CDLSTM ([48])	13.99	17.53	12.15	20.91	16.15
HMC ([49])	13.84	20.74	14.41	22.73	17.93
BiGRU-AS ([50])	13.68	20.81	15.53	27.31	19.33
DSAN ([30])	13.4	22.06	15.12	21.03	17.90
DAA ([51])	12.25	17.08	13.39	19.86	15.65
IMDSSN ([52])	12.14	17.40	12.35	19.78	15.42
BGT ([44])	12.09	**11.46**	**10.16**	**13.89**	**11.9**
CTNet ([43])	11.64	13.67	11.28	14.62	12.80
HMDAM	**10.82**	15.33	11.21	17.48	13.71

**Table 9:** Score comparison between HMDAM and other advanced methods.

Method	FD001	FD002	FD003	FD004	Average
BiLSTM ([45])	295	4130	317	5430	2543
DCNN ([46])	273.7	1041.2	284.1	12,466	5858.9
CatBoos ([47])	398.7	3493.2	584.2	3203.4	1919.9
CDLSTM ([48])	320	1758	221	2633	1233
HMC ([49])	427	19,400	2977	10,374	8295
BiGRU-AS ([50])	284	2454	428	4708	1968.5
DSAN ([30]	336	1946	251	3671	1571.3
DAA ([51])	198	1575	290	1741	951
IMDSSN ([52])	206.11	1775.15	229.54	2852.81	1265.9
BGT ([44])	262.67	**550.52**	196.94	963.36	**493.37**
CTNet ([43])	187	809	**187**	**844**	506.75
HMDAM	**170.07**	1030.42	239.47	1738.19	794.53

## 4. Conclusions and Future Research

This study proposes the HMDAM model, which extracts temporal and sensor-level features and integrates them to estimate the RUL of aircraft engines. The architecture consists of two primary modules: the TFEB, which models the evolution of time-dependent features and uncovers hidden patterns in different time scales, and the SFEB, which enhances sensor-level representation by learning channel-wise attention weights. To obtain the final RUL output, the fused features are processed through a regression block composed of dense layers and a Sigmoid function.

To assess the proposed model’s efficacy, detailed experiments were executed. A thorough investigation of sliding window configurations and model hyperparameters across the C-MAPSS sub-datasets ensured optimal performance. Ablation studies confirmed the contribution of each model component, while comparative evaluations against several state-of-the-art methods demonstrated that the HMDAM consistently achieved superior or competitive performance—particularly on simpler datasets such as FD001. In the efficiency analysis, the proposed model showed improved prediction accuracy without a substantially increased computational cost. This makes it a practical solution for RUL prediction tasks under limited computational resources.

Despite its promising results, the HMDAM model has certain limitations. Since the computational resources required by the proposed model do not markedly exceed those of the baseline, its computational accuracy may be suboptimal in complex scenarios. In addition, the model shows a tendency to overestimate RUL. For example, on the FD004 dataset, it underperformed compared with other advanced models. Furthermore, it lacks transferability, as models trained on FD001 cannot be directly applied to other sub-datasets.

In future work, we plan to enhance the robustness and generalization capability of the HMDAM by incorporating multi-scale feature fusion techniques to better capture degradation trends across diverse and complex engine states, thus, enhancing the model’s relevance and utility in practical prognostic applications.

## Figures and Tables

**Table 1:** Summary of the C-MAPSS data.

Dataset	C-MAPSS			
	FD001	FD002	FD003	FD004
Training engines	100	260	100	249
Test engines	100	256	100	248
Operating Conditions	1	6	1	6
Fault modes	1	1	2	2
Training set size	20,631	53,759	24,720	45,918
Test set size	100	259	100	218

**Table 2:** Configuration of training parameters.

Hyperparameter	Description	Option
Batch size	The number of samples for each backpropagation	32
Optimizer	Algorithm for minimizing loss	Adam
Training epochs	The number of backpropagations for each sample	100
Learning rate (lr)	Initial learning rate of training	0.001–0.0001
Dropout rate	Proportion of samples discarded	0.2

**Table 3:** Structural configuration of the proposed model.

Components	Layers	Parameters	Option
TFEB	Encoder layer	Number of Conv1d layers	2
		Kernel size of Conv1d layer	1
		Number of norm layers	2
		Number of hidden dimensions	32
		Number of extended dimensions	128
		Number of heads	12
		Activation	ReLU
		Number of encoder layers	2
SFEB	Linear network layer	Number of hidden dimensions	32
		Reduction	4
		Activation	ReLU
		Number of linear network layers	2
	Projection layer	Activation	Sigmoid
Regressor	Linear network layer	Activation	ReLU
		Number of linear network layers	3
	Prediction layer	Activation	Sigmoid

**Table 4:** Configuration of hyperparameter values.

Parameter	A	B	C	D
Number of encoder layers	1	2	3	4
Number of heads	4	8	12	16
Batch size	16	32	64	128
Model dimension	32	64	128	256

**Table 5:** Optimal hyperparameter settings for each sub-dataset.

Parameter	FD001	FD002	FD003	FD004
Number of encoder layers	3	3	3	4
Number of heads	4	16	8	16
Batch size	128	32	64	32
Model dimension	32	32	32	32

**Table 6:** Performance comparison of different methods.

Methods	FD001			FD002			FD003			FD004		
	RMSE	S-Score	MAE	RMSE	S-Score	MAE	RMSE	S-Score	MAE	RMSE	S-Score	MAE
Transformer	14.23	379.56	10.32	18.62	3751.61	12.74	12.86	326.55	10.26	22.87	9862.59	15.87
iTransformer	11.47	190.37	9.10	15.9	2499	14.08	12.32	298.08	8.97	20.09	2469	15.6
ST	12.58	273.66	9.51	17.35	2587.55	11.93	12.53	291.17	10.1	22.82	5936.06	15.89
HMDAM	10.82	170.07	9.02	15.33	1130.42	10.72	11.21	239.47	8.96	17.48	1738.19	11.88

**Table 7:** Comparison of FLOPs and parameter counts of the models.

Method	Number of FLOPs	Number of Parameters
Transformer	810.82 K	87.39 K
iTransformer	441.92 K	89.66 K
ST	1.185 M	100.50 K
HMDAM	821.25 K	89.02 K

## Data Availability

Data are contained within the article.
